# Method Development for Simultaneous Determination of 41 Pesticides in Rice Using LC-MS/MS Technique and Its Application for the Analysis of 60 Rice Samples Collected from Tehran Market 

**Published:** 2014

**Authors:** Attaollah Shakouri, Hassan Yazdanpanah, Mohammad Hossein Shojaee, Farzad Kobarfard

**Affiliations:** a *School of Pharmacy, Shahid Beheshti University of Medical Sciences, Tehran, I.R. Iran. *; b *Food and Drug Control Laboratories, Food and Drug Deputy, Iranian Ministry of Health and Medical Education, Tehran, I.R. Iran. *; c *Faroogh Life Sciences Research Laboratory, Tehran, I.R. Iran. *; d *Phytochemistry Research Center, Shahid Beheshti University of Medical Sciences, Tehran, I.R. Iran. *

**Keywords:** Pesticide residues, Multi-residue analysis, LC–MS/MS, Iran, Rice

## Abstract

A multi-residue method for simultaneous determination of 41 LC-amenable pesticides in rice, belonging to different chemical classes has been developed in Iran by LC-MS/MS. For the first time the pesticides were analyzed simultaneously in a single run using positive electrospray ionization with multiple reaction monitoring (MRM) after extraction with slightly modified QuEChERS method. The calibration curve for each analyte was linear over the concentration range of 0.02–1.0 μg/g with a correlation coefficient range between 0.993 and 0.999. The LOQ and LOD were .025 μg/g and 0.008 μg/g respectively, for all 41 pesticides and the mean recoveries obtained for three fortification levels (0.025, 0.08 and 0.250 μg/g) were 71-119% with satisfactory precision (RSD<20%). The developed method was used to investigate the occurrence of pesticides in 30 domestic and 30 imported rice samples collected from Tehran market. Five compounds were detected in 11 domestic and 9 imported positive samples in concentration range from 0.032 μg/g to 0.081 μg/g and 0.028 μg/g to 0.074 μg/g, respectively. With the exception of prohibited pesticides, phosphamidon and TCMTB, three permitted pesticides, cinosulfuron, triadimenol and tricyclazole, found in positive rice samples were below MRLs established by Institute of Standards and Industrial Research of Iran (ISIRI).

## Introduction

Pesticides consist of a large number of substances that are applied to crops at various stages of cultivation to provide protection against pests and during post-harvest storage to preserve quality. More than 1000 active pesticide ingredients have been employed and are currently formulated in thousands of different commercial products. These chemicals, as well as their metabolites, show very different physico-chemical characteristics and large differences in polarity, volatility and persistence (1). Pesticides are chemically completely heterogeneous group of compounds such as organochlorines, organophosphorates, carbamates, pyrethroids and substituted ureas and are used as fungicides, acaricides, insecticides, herbicides, *etc*., in soils and cultivations treatments (2). 

They are in significant public benefit by increasing agricultural productivity as was demonstrated in the green revolution in less developed countries, and by decreasing the prevalence of diseases. However, they also cause public concern due to their potential adverse effects on human health, which is most obvious in the developing fetus and young child (3). The most common route of exposure to pesticides is by ingestion of treated food commodities containing residues. Most pesticide residues occur in food as a result of the direct application of a pesticide to a crop or farm animal or the post-harvest treatments of food commodities. To ensure the safety of food for consumers, numerous legislations such as the EU directives (4) or the Iranian regulation (5) have established maximum residue limits (MRL) for pesticides in foodstuffs. Therefore, control and management of pesticide residues in foods, according to regulations require powerful analytical methods. 

Analytical methodologies employed must be capable of residue measurement at very low levels and must also provide unambiguous evidence to confirm both the identity and the concentration of any residue detected (6-7).

For the monitoring of pesticides, gas chromatography (GC) with electron capture detection (ECD), nitrogen–phosphorus detection (NPD) and mass spectrometry (MS) detection have been the most widely used techniques for many years. However, many pesticides which are thermally unstable or non-volatile such as carbamates or benzimidazoles are difficult or impossible to analyze using these classical GC and GC–MS techniques. Liquid chromatography (LC) coupled to tandem mass spectrometry offers a powerful tool for the determination of these compounds in food samples (8). One analytical challenge involved in pesticide-residueanalys is is that matrix components co-extracted with pesticides produce several additional signals in chromatograms that can lead to false-positive identifications. While such interferences are not odd if extracts of complex matrices (*e.g*., herbs or tea) are analyzed by GC-MS or by LC-MS using selected ion monitoring (SIM), this is not so when MS/MS is used (9). For this reason, LC-MS/MS methods do not require either extensive clean-up or sophisticated chromatographic separation. Different molecules that share the same transition are found more rarely than molecules producing fragments of identical mass. As a consequence, peak identification is easier and faster in LC-MS/MS than in GC-MS or LC-MS (10).

Besides powerful instruments, determination of pesticide residues in food requires exact extraction procedures. Recently, various extraction procedures have been applied in pesticide residues analysis using acetone, ethylacetate and acetonitrile solvents in fruit and vegetables (11). In 2003, Anastassiades *et al. *reported an acetonitrile based method for sample preparation called as QuEChERS (Quick, Easy, Cheap, Effective, Rugged and Safe). This method covers a very wide scope of analytes, including polar, semi-polar and non-polar pesticide residues in various food matrices. The procedure involves initial single-phase extraction of the sample with acetonitrile, followed by liquid–liquid partitioning by the addition of anhydrous magnesium sulfate (MgSO_4_) and sodium chloride. Removal of water and clean-up are performed simultaneously on an aliquot of the acetonitrile extract with dispersive solid phase extraction using MgSO_4_ and primary secondary amine (PSA) sorbent(12). Nowadays, combination of the QuEChERS method and chromatography-mass spectrometry (LC/MS and GC/MS) instruments have been successfully employed to determine multi-residue pesticides in different food matrices, including rice samples (13- 22).

Rice is one of the most consumed foods in the world, including in Iran. The consumption of rice in Tehran is 110 g per capita/day (Personal Communication). Rice consumption has increased in the recent decades, with a consequent rise in the use of pesticides to improve its production yield, like pre and post-emergence herbicides, insecticides, and fungicides during various stages of cultivation (23). The use of these pesticides affects the whole system of rice: the soil, water, and rice grain. In addition to commonly used pesticides, presence of banned pesticides in rice is another important challenge. For these reasons there is a clear need to develop fast methods for the multi-residue analysis of the most commonly used and forbidden pesticides in rice crops.

In the present work, a multi-residue method for determination of pesticide residues in rice, using modified QuEChERS method (12) and determination by means of LC–ESI–MS/MS, is introduced for the first time in Iran. The validated method is then applied for determination of 41 pesticide residues, including 18 allowable and 23 banned pesticides in 30 domestic and 30 imported rice samples (total 60 samples) collected from Tehran market.

## Experimental

*Chemicals*

Pesticides reference standards (purity > 96.0%), triphenylphosphate (TTP), as internal standard and anhydrous magnesium sulfate (MgSO_4_), were purchased from Sigma–Aldrich /Fluka /Riedel-de-Haën (Germany). Ammonium formate, methanol (MeOH) and HPLC-grade acetonitrile (MeCN) were purchased from Across (Belgium). Ethyl acetate (EtAc), glacial acetic acid (HOAc) and sodium acetate were supplied from Merck (Darmstadt, Germany). Bondesil-primary secondary amine (PSA, 40 μm) was provided from Interchim (France). HPLC grade water was obtained by purifying demineralized water on a Milli-Q Plus ultra-pure water system (Millipore, Molsheim, France).

Individual stock solutions of the pesticides at a concentration of 1000 μg/mL were prepared in ethyl acetate and methanol (Cartap and Fuberidazole) according to their solubility at 20 ^◦^C. A mixed intermediate standard solution at a concentration of 5 μg/mL was prepared via appropriate dilution of the stock solutions in MeOH containing 0.1% HOAc in order to avoid the degradation of the pesticides (8). This solution was used as a spiking solution for validation experiments. Matrix-matched multi-level calibration standards solutions were prepared in sample extracts obtained from organic rice. Aliquots of blank samples (5 mL of final MeCN layer), which were extracted via QuEChERS method were evaporated and reconstituted in 5 mL of mixture of appropriate working standard solutions and 0.02% HOAc in MeOH to generate final concentrations of 0.02, 0.04, 0.10, 0.20, 0.50 and 1.0 μg/g for the matrix-matched calibration standards. A stock solution of triphenylphosphate (TTP) in ethyl acetate at concentration of 20 μg/mL was used as internal standard and an aliquot of 50 μL of TTP solution in ethyl acetate (20 μg/mL) was added to the spiked rice sample as internal standard. 

*Pesticide selection*

The 18 selected LC-amenable pesticides (carbaryl, cartap, chlorpyrifos, cinosulfuron, diazinon, edifenphos, malathion, oxadiazon, oxydemeton-methyl, primiphos-methyl, propiconazole, spynosin A and D, thiobencarb, thiophanate-methyl, triadimenol, tricyclazole, triflumizole) are used for rice production in Iran and MRL_s_ have been established for them by Institute of Standards and Industrial Research of Iran (ISIRI), NO.13120 (5). According to the same act some of pesticides are forbidden to use in Iran. Existence of banned pesticides in any kind of food including rice can produce health problems and it is necessary to investigate the presence of them in foods. Therefore, 23 banned LC-amenable pesticides according to ISIR̕s list (5), including; azinphos-ethyl, bromacil, carbofuran, chlorbromuron, chlorfenvinphos, coumaphos, dialifos, dicrotophos, etrimfos, fluometuron fuberidazole, iprobenfos, methabenzthiazuron, methidathion, monocrotophos, omethoate, phosphamidon, phoxim, propoxur, pyrazophos, TCMTB, tri-allate, triazophos were selected. 

The comprehensive list covers 41 pesticides with different modes of action such as herbicides, fungicides, insecticides and plant growth regulators with different chemical natures such as organophosphates, carbamates, micro-organism derived (Spinosyn), strobilurins and quarternary ammoniums. 

*Rice samples*

Sixty rice samples, including 30 domestic and 30 imported samples were collected from different regions of Tehran in early six months of 1388 (March 23- September 23, 2009). A 100 grams portion of the collected samples were grinded with 100 g dry ice, right after purchase and stored in -70 ^°^C until analysis. 

*Liquid chromatography*

The separation of the different pesticides from the samples was carried out using an Alliance separations module 2695 (Waters, Milford, MA, USA), which consist of a quaternary solvent delivery system, degasser, autosampler, column heater and diode array detector coupled with a Quattro Micro Triple Quadrupole LC/MS (Waters, Micromass, Manchester, UK).

Chromatographic separation was performed using an Agilent ZORBAX Eclipse XDB-C_18 _(Narrow-Bore 2.1×150 mm, 3.5-micron) analytical column at a flow rate of 200 μL/min and an injection volume of 100 μL. The mobile phase was 5 mM in methanol (solvent A) and 5 mM ammonium formate in water (solvent B) in a ammonium formate gradient mode and a total analysis time of 30 min. The elution program was as follows: at the start 30% solvent A and 70% solvent B; the percentage of solvent A was linearly increased to 100% in 20 min, then remained constant for 5 minand ramped to original composition in 5 min. The temperature of the column heater was maintained at 40 °C.

*Mass spectrometry*

The MS/MS system consisted of a triple quadrupole mass spectrometer Quattro Micro (Waters-Micromass, UK) equipped with an electrospray source (Z-spray) and operated in positive ionization mode. Mass Lynx software, version 4.0, was used for instrument control and data acquisition. Analysis was performed in positive ion mode. The ESI source values were: capillary voltage, 4.12 kV; extractor, 2 V; RF lens, 0.1 V; source temperature, 120 ◦C; desolvation temperature, 300 °C; desolvation gas and cone gas (nitrogen 99.99% purity) flow rates, 500 and 50 L/h, respectively. The analyzer settings were: resolution, 14.6 (unit resolution) for LM1 and LM2 resolution and 14 for HM1 and HM2 resolution; ion energy1 and 2, 0.3 and 3.0, respectively; entrance and exit energies, 55 and 75 (V); multiplier, 700 (V); collision as (argon, 99.995%) pressure 5.35 × 10^-3^ mbar.

*Sample preparation*

Extraction was performed by the original QuEChERS method (12). Five g of homogenized rice sample was accurately weighed into a 50 mL centrifuge tube. Appropriate concentrations of the mixed working standard solution (for spiking) and internal standard were added to the tube and 10 mL of acetonitrile (MeCN) was added. The mixture was vortex mixed for 2.0 min, followed by addition of a mixture of 2 g anhydrous MgSO_4_ and 1.5 g sodium acetate and vortex mixing for 2.0 min again. The mixture was centrifuged for 5 min at 5433×g, and 5 mL of the supernatants was then transferred into an appropriate tube containing 100 μL methanol (MeOH) placed in a nitrogen evaporator and evaporated at 40 °C until dryness.The residue was reconstituted in 5 mL MeCN. The mixture was vortex mixed for 2.0 min followed by sonication for 4.0 min and the solution was transferred to atube containing 60 mg anhydrous MgSO_4_ and 20 mg PSA (primary secondary amine). The mixture was vortex mixed vigorously for 2 min and centrifuged for 5 min at 5433×g. Finally, a 5 mL aliquot of the cleaned extract was transferred into a screw cap vial and 100μL of the solution was injected into LC-MS/MS. 

*Method validation*

The validation study was performed based on the European SANCO guidelines (24). The method was tested to assess for sensitivity, mean recovery, precision, and limit of quantification (LOQ). This requires performing recovery experiments with spiked blank samples to estimate the accuracy of the method. A minimum of 5 replicates is required (to check the precision) at both the reporting limit (to check the sensitivity of the method), and at least another higher level. 

Linearity was studied using matrix-matched calibrations by analyzing in triplicate six concentration levels, between 0.01 and 1.0 μg/g.

For determination of mean recoveries and precision (repeatability, expressed as coefficient of variation (in %), five spiked blank rice samples at concentration levels of 0.025, 0.08 and 0.250 μg/g were prepared and then treated according to the procedure described in sample preparation. The recoveries were calculated using the matrix-matched calibration.

The limit of quantification (LOQ) was established as the lowest validated spike level meeting the method performance acceptability criteria (mean recoveries for each representative commodity in the range 70-120%, with an RSD ≤ 20%).

The concentrations of pesticides were determined by interpolation of the relative peak areas for each pesticide to internal standard peak area in the sample on the spiked calibration curve. In order to compensate for losses during sample processing and instrumental analysis, internal standard (TPP) was used.

## Results and Discussion

*LC- MS/MS determination*

All of the pesticides under study were optimized in the positive electrospray ionization (ESI +) mode and multiple reaction monitoring (MRM) experiments were conducted with a dwell time and inter-channel delay of 0.06 and 0.1s, respectively. The optimization of the precursor ion, product ions, cone voltageand collision energy was performed via direct injection of the individual pesticide standard solution (1 μg/mL) into the mass spectrometer using a syringe pump at flow rate 10 μL/min. The most intense transition was used for quantitation, while the other was employed for confirmation. The optimized parameters are presented in Table 1. Acquisition was conducted in 8 acquisition functions.

**Table1:** Summary of molar masses, MRM parameters, ion ratios and retention time data for analysis of the pesticides in ESI, positive mode

**No**	**Pesticides**	**Molar mass**	**Precursor ion**	**CV(V)**	**1st Transition (quantitation)**	**CE (eV)**	**2nd Transition (confirmation)**	**CE(eV)**	**Rt(min)**	**Ion ratio**
1	Azinphos-ethyl	345	[M+H]^+^	15	346⟶77	36	346⟶132	30	18.99	1.43
2	Bromacil	261	[M+H]^+^	20	261⟶205	12	261⟶188	35	13.75	7.03
3	Carbaryl	201	[M+H]^+^	15	202⟶145	20	202⟶117	10	14.61	3.85
4	Carbofuran	221	[M+H]^+^	15	222⟶165	16	222⟶123	16	15	1.4
5	Cartap	237	[M+H]^+^	27	238⟶73	16	238⟶150	16	2.57	1.75
6	Chlorbromuron	292	[M+H]^+^	28	293⟶204	16	293⟶182	16	18.47	1.3
7	Chlorfenvinphos	358	[M+H]^+^	28	359⟶99	28	359⟶155	17	21.05	2.11
8	Chlorpyrifos	350	[M+H]^+^	30	350⟶97	25	350⟶198	22	25.24	2.08
9	Cinosulfuron	413	[M+H]^+^	16	414⟶183	15	414⟶157	15	5.67	17.9
10	Coumaphos	362	[M+H]^+^	35	363⟶307	17	363⟶289	25	20.95	3.23
11	Dialifos	393	[M+H]^+^	20	394⟶187	10	394⟶208	15	22.77	1.12
12	Diazinon	304	[M+H]^+^	29	305⟶97	35	305⟶169	20	21.92	1.88
13	Dicrotophos	237	[M+H]^+^	26	238⟶112	10	238⟶193	10	4.32	3.34
14	Edifenphos	310	[M+H]^+^	30	311⟶109	32	311⟶111	26	20.5	4.85
15	Etrimfos	292	[M+H]^+^	35	293⟶125	25	293⟶265	18	21.67	2.66
16	Fluometuron	232	[M+H]^+^	30	233⟶72	18	233⟶46	20	15.33	4.87
17	Fuberidazole	184	[M+H]^+^	42	185⟶157	25	185⟶156	32	12.21	2.24
18	Iprobenfos	288	[M+H]^+^	20	289⟶91	18	289⟶205	14	20.19	6.68
19	Malathion	330	[M+H]^+^	18	331⟶127	12	331⟶99	20	18.43	1.44
20	Methabenzthiazuron	221	[M+H]^+^	28	222⟶165	20	222⟶150	30	15.53	2.16
21	Methidathion	302	[M+H]^+^	18	303⟶145	20	303⟶85	10	16.93	1.23
22	Monocrotophos	223	[M+H]^+^	26	224⟶127	18	224⟶98	14	3.73	2.25
23	Omethoate	213	[M+H]^+^	20	214⟶125	18	214⟶183	15	3.1	4.35
24	Oxadiazon	344	[M+H]^+^	30	345⟶220	13	345⟶177	40	24.27	1.49
25	Oxydemeton-methyl	246	[M+H]^+^	20	247 ⟶109	25	247 ⟶169	14	3.26	1.28
26	Phosphamidon	299	[M+H]^+^	26	300⟶127	20	300⟶174	10	12.47	2.71
27	Phoxim	298	[M+H]^+^	16	299⟶129	13	299⟶153	11	21.46	5.91
28	Primiphos-methyl	305	[M+H]^+^	30	306⟶108	28	306⟶164	17	22.44	4.76
29	Propiconazole	341	[M+H]^+^	40	342⟶159	30	342⟶69	16	21.26	1.04
30	Propoxur	209	[M+H]^+^	20	210⟶111	14	210⟶168	8	13.93	3.23
31	Pyrazophos	373	[M+H]^+^	36	374⟶222	30	374⟶194	21	22.13	1.3
32	Spinosyn A	732	[M+H]^+^	53	733⟶142	30	733⟶98	56	26.65	3.72
33	Spinosyn D	746	[M+H]^+^	50	747⟶142	31	747⟶98	51	27.44	4.36
34	TCMTB	238	[M+H]^+^	21	239⟶180	10	239⟶136	40	18.1	3.6
35	Thiobencarb	257	[M+H]^+^	18	258⟶125	20	258⟶100	13	22.57	7.66
36	Thiophanate-methyl	342	[M+H]^+^	24	343⟶151	22	343⟶311	12	13.51	6.06
37	Triadimenol	295	[M+H]^+^	20	296⟶70	8	296⟶99	15	19.66	7.43
38	Tri-allate	303	[M+H]^+^	32	304⟶86	20	304⟶143	24	25.34	1.08
39	Triazophos	313	[M+H]^+^	31	314⟶162	18	314⟶119	32	18.85	1.8
40	Tricyclazole	189	[M+H]^+^	38	190⟶136	27	190⟶163	22	10.4	1.41
41	Triflumizole	345	[M+H]^+^	10	346⟶278	8	346⟶73	25	23.17	2.36
42	Triphenylphosphate*	326	[M+H]^+^	20	327⟶77	45	327⟶152	45	20.81	1.94

* Internal standard

The initial liquid chromatography (LC) method, developed in our laboratory, was setup using a methanol and water gradient composition. This mobile phase composition gave very poor response for most pesticides. Therefore, it was decided to reconsider the LC mobile phase conditions. Hence, methanol containing 0.1% formic acid and water combination was used. In this condition, some pesticides gave better response but it was noticed that formation of sodium adducts as a new challenge, suppressed some pesticide responses. Another mobile phase compositions that was examined to optimize responses of the pesticide in the LC–MS/MS system was MeCN and water. Finally, the gradient profile of 5-mM ammomium formate in methanol and 5-mM ammonium formate in water gave the overall best result. Pesticides exhibiting insufficient response in this experiment were tuned in the LC–MS/MS system for optimum conditions. With the ammonium formate buffer, the presence of ammonium ions suppressed the formation of sodium adducts, which are more common under acidic conditions, and therefore, pesticides formed [M+H]^+^, which showed higher sensitivity and more consistent responses for certain pesticides. Pesticides were identified according to their retention times, target and qualifier ions. The quantitation was based on the peak area ratio of the targets to that of internal standard. Table 1 summarizes pesticides studied with their target and qualifier ions used in the MRM mode in this study.

*Method validation*

The recovery results obtained from analysis of rice samples are shown in Table 2. The pesticides showed good linearity in the MRM mode. Linear matrix-match calibration curves for all the pesticides were obtained with a correlation coefficient range between 0.993 and 0.999. 

The recovery of pesticides at three spike levels (0.025, 0.08 and 0.250 μg/mL) was in the range of 71-119%. In terms of repeatability, all pesticides gave RSD <20% with n = 5 at each spiking level. The recoveries and repeat abilities are in accordance with the criteria set by SANCO Guideline (Commission of the European Communities) (24).

Limits of quantification (LOQs) of the proposed method were calculated using SANCO Guideline where the lowest spiked concentration that gave mean recovery 70-120% and RSD<20, therefore, LOQ of the method was 0.025 μg/g. LOD of the method was calculated by LOQ/3 and was 0.008 μg/g.

**Table 2:** Average recoveries (%) and relative standard deviations, RSD (%), obtained for 41 pesticides in rice samples, spiked at 0.025, 0.08 and 0.25 μg/g levels (n=5).

933 **NO.**	**Pesticides **	**0.025 μg/g**		**0.08 μg/g**		**0.250 μg/g**		
		**Recovery**	**RSD**	**Recovery**	**RSD**	**Recovery**	**RSD**
1	Azinphos-ethyl	87	11	84	9	99	10	
2	Bromacil	109	6	93	10	97	8	
3	Carbaryl	98	4	110	5	98	9	
4	Carbofuran	86	5	97	6	105	13	
5	Cartap	112	4	94	5	91	9	
6	Chlorbromuron	101	16	105	17	89	17	
7	Chlorfenvinphos	84	7	102	4	97	10	
8	Chlorpyrifos	111	8	92	11	93	18	
9	Cinosulfuron	84	9	99	6	90	10	
10	Coumaphos	108	19	94	15	92	16	
11	Dialifos	113	4	77	8	87	10	
12	Diazinon	109	4	71	8	85	9	
13	Dicrotophos	108	18	79	11	95	15	
14	Edifenphos	75	9	93	18	85	11	
15	Etrimfos	107	4	97	8	94	10	
16	Fluometuron	79	16	96	18	72	11	
17	Fuberidazole	114	5	97	7	106	10	
18	Iprobenfos	97	7	82	6	84	9	
19	Malathion	94	4	96	9	86	10	
20	Methabenzthiazuron	107	8	112	7	104	12	
21	Methidathion	113	6	97	12	89	17	
22	Monocrotophos	94	5	94	9	93	10	
23	Omethoate	84	5	100	9	111	10	
24	Oxadiazon	110	6	89	8	104	11	
25	Oxydemeton-methyl	106	6	108	16	88	10	
26	Phosphamidon	109	5	98	9	95	6	
27	Phoxim	88	18	93	9	81	15	
28	Primiphos-methyl	103	6	97	6	93	10	
29	Propiconazole	110	4	94	11	87	6	
30	Propoxur	81	6	96	10	95	13	
31	Pyrazophos	108	8	97	16	95	14	
32	Spinosyn A	102	10	100	17	104	19	
33	Spinosyn D	96	5	94	7	101	13	
34	TCMTB	119	6	89	10	92	18	
35	Thiobencarb	105	4	92	12	99	9	
36	Thiophanate-methyl	75	13	90	19	100	16	
37	Triadimenol	80	4	91	12	86	10	
38	Tri-allate	80	7	93	7	95	8	
39	Triazophos	106	5	100	10	95	13	
40	Tricyclazole	92	7	102	8	93	15	
41	Triflumizole	73	6	90	13	80	11	
42	Triphenylphosphate*	98	13	88	7	90	10	

* Internal standard

*Application of the method to real samples*

The method was applied for the analysis of 60 real rice samples, including 30 domestic and 30 imported collected from different local markets of Tehran located in twenty two regions in early six months of 1388 (March 23- September 23, 2009). As shown in table 3, among the 41 pesticides, 5 compounds were found in both domestic and imported positive samples. In domestic samples, cinosulfuron was the most common pesticide residue detected (found in 17% of samples), followed by triadimenol and tricyclazole both detected in 6% of samples, and phosphamidon, TCMTB(1% of domestic samples). 

In imported rice samples, TCMTB was the most common pesticide residue detected (10% of samples), followed by cinosulfuron and triadimenol (2% of imported samples) and phosphamidon and tricyclazole (1% of imported samples).

Using of three pesticides cinosulfuron, triadimenol and tricyclazole are allowed in Iran for rice production and the concentrations found for them are below MRL_s_ established by ISIRI. According to the ISIRI̕s regulations, phosphamidon and TCMTB are forbidden in Iran and their presence in the samples is worth the attention.

**Table 3:** Numbers and concentration ranges of pesticides found in domestic and imported rice samples analyzed with ISRIʾs MRL (μg/g).

**No.**	**Pesticides**	**No. of positive samples**		**Concentration range min- max (μg/g)**		**ISRIʾs MRL (μg/g)**
		**Domestic**	**Imported**	**Domestic**	Domestic	**Imported**
1	Cinosulfuron^a^	5 (17%)	2 (6%)	0.032-0.041	0.028-0.045	0.05
2	Phosphamidon^b^	1 (3%)	1 (3%)	0.039	0.044	
3	TCMTB^b^	1 (3%)	3(10%)	0.047	0.031-0.050	
4	Triadimenol^a^	2 (6%)	2 (6%)	0.043-0.066	0.030-0.058	0.5
5	Tricyclazole^a^	2 (6%)	1 (3%)	0.056-0.081	0.074	5.0

a . Permitted pesticides in Iran for rice production.

b . Prohibited pesticides in Iran.

## Conclusions

For the first time in Iran, an accurate, precise, sensitive and selective multi-residue method was developed for the simultaneous detection, quantification and confirmation of 41 pesticide residues (belonging to very different chemical families) in rice using QuEChERS sample preparation procedure and LC–MS/MS. The validation results have shown excellent recoveries (71-119%) and precision (RSDs <20%) for all pesticides studied, meeting EU guidelines method performance criteria. The method was applied successfully for the analysis of 60 real samples. Five compounds were found in positive samples. Twenty samples were contaminated with cinosulfuron, triadimenol and/or tricyclazole at the levels below Iranian maximum residue limits (MRLs) in rice. For other detected pesticides, no MRLs have been set in rice in Iran. Therefore, 6 out of 60 (10 %) samples were contaminated with illegal pesticides.
